# Development of ARPE-19-Equipped Ocular Cell Model for In Vitro Investigation on Ophthalmic Formulations

**DOI:** 10.3390/pharmaceutics15102472

**Published:** 2023-10-16

**Authors:** Simona Sapino, Giulia Chindamo, Elena Peira, Daniela Chirio, Federica Foglietta, Loredana Serpe, Barbara Vizio, Marina Gallarate

**Affiliations:** 1 Department of Drug Science and Technology, University of Turin, 10125 Turin, Italy; giulia.chindamo@unito.it (G.C.); daniela.chirio@unito.it (D.C.); federica.foglietta@unito.it (F.F.); loredana.serpe@unito.it (L.S.); marina.gallarate@unito.it (M.G.); 2 Department of Medical Sciences, University of Turin, Via Genova 3, 10126 Turin, Italy; barbara.vizio@unito.it

**Keywords:** in vitro ocular flow cell, intravitreal injections, ARPE-19 scaffold, bevacizumab, drug delivery systems

## Abstract

Repeated intravitreal (IVT) injections in the treatment of retinal diseases can lead to severe complications. Developing innovative drug delivery systems for IVT administration is crucial to prevent adverse reactions, but requires extensive investigation including the use of different preclinical models (in vitro, ex vivo and in vivo). Our previous work described an in vitro tricompartmental ocular flow cell (TOFC) simulating the anterior and posterior cavities of the human eye. Based on promising preliminary results, in this study, a collagen scaffold enriched with human retinal pigmented epithelial cells (ARPE-19) was developed and introduced into the TOFC to partially mimic the human retina. Cells were cultured under dynamic flow conditions to emulate the posterior segment of the human eye. Bevacizumab was then injected into the central compartment of the TOFC to treat ARPE-19 cells and assess its effects. The results showed an absence of cytotoxic activity and a significant reduction in VEGF fluorescent signal, underscoring the potential of this in vitro model as a platform for researching new ophthalmic formulations addressing the posterior eye segment, eventually decreasing the need for animal testing.

## 1. Introduction

Treating retinal diseases with injections into the vitreous body is a common practice in the ophthalmic clinic. Repeated IVT administration is part of the treatment of certain diseases such as age-related macular degeneration (AMD) and diabetic macular edema (DME). However, in addition to being unpleasant and a reason for low patient compliance, IVT injections can lead to complications, such as retinal detachment, vitreous hemorrhage, and endophthalmitis [1].

The development of novel therapeutic systems that are able to release drugs in a delayed manner is therefore one of the most important goals in the field of ophthalmology. As a result of the increased permanence of the drugs in the posterior segment of the eye, fewer administrations would be required, making IVT therapies less invasive, more harmless, and more comfortable for the patients.

As with all new pharmaceutical preparations, the study of novel ophthalmic therapeutic systems involves the use of in vitro and in vivo experimental models in the preclinical phase [2]. In fact, once a candidate pharmaceutical formulation is identified, its development follows a detailed plan to ensure both safety and efficacy.

To date, preclinical studies for new preparations have been dominated by animal models. However, despite their importance, several efforts are underway at present to reduce the number of animals used in testing, both to comply with ethical concerns and to reduce costs. In addition, humans and animals differ in many ways, which is another concern with the employment of animal testing. This is especially true for experiments in ophthalmic research, in which animal models with intact vitreous humor are often used, which do not take into account structural changes induced by disease, age-related differences in vitreous humor, and interspecies variability (as an example, the volume of the rabbit vitreous is only around 1.5 mL, while it is 3–4 mL for humans).

The use of animals in medical and scientific research has recently been the subject of heated debate, and with recent changes to European legislation, specific funds have been allocated to research on alternative methods [3]. In this context, in vitro models as alternatives to animal testing become important, as they can speed up the course of medicinal product development and optimization while decreasing costs and reducing animal use [4,5]. Moreover, these alternative models can also decrease the huge gap between in vitro and in vivo studies, offering an alternative and/or support before moving to clinical trials [6,7]. Among alternative in vitro approaches, three-dimensional (3D) cell cultures recreate in vivo tissue microenvironments, and are able to increase cell–cell/matrix interactions, along with mechanical and chemical properties of tissue-like structures, compared to classic two-dimensional (2D) cell cultures [7,8]. Specifically, alternative models based on the seeding and culture of cells within porous 3D scaffolds composed of different materials with potentially tunable architectural complexity have also been recently described [8]. The current trend is to build bottom-up models that include anatomical and physiological factors, pharmacokinetic parameters, and consider physicochemical characteristics of drugs. These models are called pharmacokinetic models (PK models). Accordingly, some researchers have developed models to predict drug fate in the vitreous to accelerate the development and optimization of therapeutic dosage forms in the preclinical phase [9,10].

To quantify and predict the efficiency of drug delivery, it is essential to have in vitro models that mimic the human eye in terms of compartmentalization and mass transport processes that occur in vitreous humor. Indeed, some researchers [11] presented an experimental study of vitreous motion induced by saccadic eye movements, employing a model in which care was taken to correctly reproduce real saccadic eye movements.

Interestingly, an in vitro eye model (PK-EyeTM) has been developed to estimate human pharmacokinetics of IVT therapeutic compounds and formulations, which are cleared through the anterior route [12]. This model, designed with dimensions similar to the human eye, has proven to be highly effective in evaluating the clearance profiles of proteins like bevacizumab (BVZ) and ranibizumab, as well as poorly soluble drugs such as triamcinolone acetonide when administered as suspensions or formulated as IVT implants [13]. However, it does not provide insights into the tissue absorption and distribution of these injected formulations.

Another in vitro test described in the literature combines a vitreous model [14] and a simple system described by Loch and colleagues [15] in an attempt to create an in vitro system resembling the vitreous body and the applied forces that move the depot [16]. This model, called EyeMoS [17], is composed of a spherical eye chamber, obtained by 3D printing, housed on a rotating device and filled with gel of polyacrylamide, coated in turn with a thin layer of agarose gel. The chamber is provided with an inlet channel (for injection) and an outlet channel. However, this model presents a single chamber, and has no semi-permeable barriers or supports for line cells, limiting its use.

Some patented eyeball models are already available for ocular studies. This is the case for the “in vitro eyeball superfusion system” [18], which is a device able to preserve the normal morphology of an in vitro eyeball and maintain its ability to respond to stimuli. It comprises the in vitro eyeball storing unit and a thermostatic water feeding mechanism which drips a liquid with specific ion concentration on the surface of the isolated eyeball according to a certain speed to maintain the moisture state of a cornea.

An additional example is the “artificial eye assembly”, which consists of layered components that offer pressurized multi-modular chambers. This assembly is valuable for investigating ocular pharmacokinetics while considering various ocular parameters and physiological conditions. Each chamber within the artificial eye can be pressurized at different levels, enabling the simulation of different wake and sleep cycles [19]. However, it should be noted that this artificial eye assembly does not include a compartment suitable for serving as a scaffold for retinal cell lines. Consequently, it is not suitable for conducting studies on the interaction between drugs and retinal cell lines.

Moreover, the “medical simulation human eye simulation module” [20] and “methods and devices for modeling the eye” have also been developed [21]. The former is a model for simulating the blinking and the pupil contracting mechanism, while the second is a biomimetic model of the eye comprising a convex scaffold, a fluidic device, and a fabricated eyelid coupled to a motor. In certain embodiments, the scaffold can be impregnated with one or more layers of keratocytes and epithelial cells. However, both of these models cannot be used for simulating IVT injections.

Starting from the above cited literature, particularly from the PK-EyeTM model [12], we designed and developed a Plexiglas tricompartmental eye flow cell (TOFC) that recreates the anterior and the posterior cavities of the human eye. As described in our previous paper [22], this artificial ocular model enables the simulation of IVT injections, the estimation of drug residence time in the posterior segment, and evaluation of its drainage through the anterior chamber of the eye. In the present paper, an evolution of such tricompartmental flow cells is presented, consisting of the insertion of a collagen scaffold enriched with human retinal cells mimicking the retina and allowing tests of cytotoxicity and efficacy.

For this purpose, a three-dimensional (3D) model of a human pigmented retinal (ARPE-19) cell seeded over a collagen porous scaffold has been introduced into the ocular cell and grown under a dynamic flow, in order to emulate the condition of the posterior segment of the human eye. The scaffold serves as a framework for the cells and emulates the natural extracellular matrix, allowing the assessment of interactions between the retinal cells and the drug being tested in a biologically relevant context.

The primary objective of this study is to evaluate the feasibility and practicality of this evolutionary approach. For these reasons, the viability of ARPE-19 cells over the 3D scaffold was initially evaluated under both dynamic and static conditions, the latter serving as a control. Subsequently, an analysis was conducted on a commercial solution of BVZ to examine its clearance profile and investigate its biological activity, particularly its anti-VEGF (vascular endothelial growth factor) properties. This comprehensive evaluation provides valuable insights into the behavior of retinal cells and the tested formulation, contributing to the overall understanding of the potential and applicability of this in vitro model.

## 2. Materials and Methods

### 2.1. Materials

Agar was from Alfa-Aesar (Ward Hill, MA, USA); hyaluronic acid sodium salt 1600 kDa (HA) was from Farmalabor (Barletta, Italy). Phosphate-buffered saline (PBS); Dulbecco’s Modified Eagle Medium/Nutrient Mixture F-12 (DMEM-F12) fetal calf serum (FBS); sodium azide; L-glutamine; penicillin and streptomycin; MTT solution; and propidium iodide (PI) were from Sigma-Aldrich (St. Louis, MO, USA). Avastin^®^ (Roche, Basilea, Switzerland) was kindly provided by Molinette Central Hospital (Turin, Italy). Tissue culture flasks and plates were from TPP, Trasadingen, Switzerland. Collagen porous scaffold was from Ultrafoam, Avitene; Davol Inc., Warwick, RI, USA. ARPE-19 cell line (ATCC-CRL-2302) was from ATCC^®^ (Manassas, VA, USA). Collagenase IV was from Worthington Biochemical Corporation, Lakewood, NJ, USA. VEGF (JH121); sc-57496 was from Santacruz, DBA Italia SRL, Milan, Italy. Goat anti-mouse IgG H&L (Alexa Fluor^®^ 647) ab150119 was from Abcam, Milano, Italy.

### 2.2. Methods

#### 2.2.1. Samples Preparation

Avastin (25 mg/mL BVZ) was used undiluted for clearance study. However, for cytotoxicity experiments and anti-VEGF activity assessments, it was appropriately diluted in DMEM-F12 to achieve a concentration of 1 mg/mL BVZ.

An artificial fluid simulating the humor vitreous (HV) was prepared according to the literature [23]. Firstly, agar (0.4 g) was dissolved in 100 mL of pH 7.4 PBS or DMEM-F12 medium and then carefully vortexed using IKA T-25 Ultraturrax (IKA^®^-Werke GmbH, Staufen Germany). Separately, 1600 kDa hyaluronic acid sodium salt (HA) (0.5 g) was dispersed in 100 mL water. Equal volumes of both solutions were then mixed for 5 min using Ultraturrax to obtain a homogeneous medium to which a few drops of 0.02% *w*/*v* sodium azide were added. The mixture was cooled to room temperature (20 °C) until a gel-like consistency was reached.

The simulated HV prepared in DMEM-F12 medium was sterilized by autoclaving at 121 °C for 20 min, cooled down, and then enriched with fetal calf serum (10%, *v*/*v*), L-glutamine (2 mM), penicillin (100 units/mL), and streptomycin (100 µg/mL) before being used.

#### 2.2.2. Clearance Study

**Figure 1:**
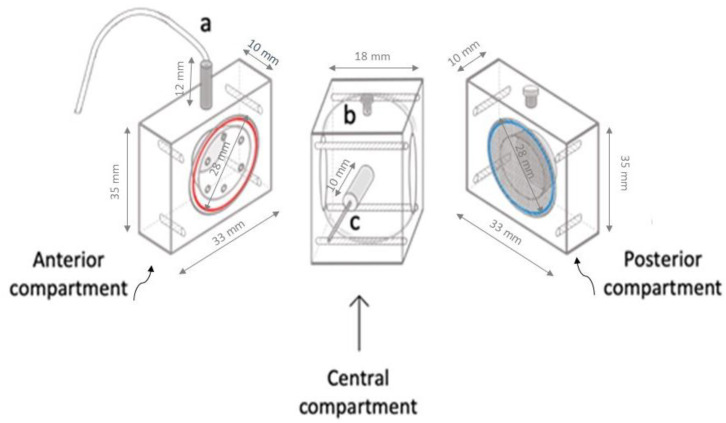
Schematic representation of the Plexiglas TOFC before assembly. The anterior compartment, on the left, features an outlet port located at the top (a) and a semipermeable disk (highlighted in red). The central compartment, in the middle, includes an injection port (b) and an inlet port (c). The posterior compartment, on the right, is equipped with a grid (highlighted in blue) that can be replaced with a retinal cell scaffold.

To study the clearance profile of BVZ, the proposed Plexiglas model (TOFC) was set up without a scaffold, as previously described [22]. Figure 1 depicts the three compartments before assembly, with the anterior compartment containing a small cavity (0.25 mL) that mimics the anterior chamber of the eye, and the central compartment with a cavity volume of approximately 7.75 mL, which simulates the vitreous body. Additionally, two disks can be observed, highlighted in blue (anterior) and red (posterior), which simulate the physiological semipermeable barriers, namely the uveal trabecular and the retina, respectively. The anterior disk is designed with eight microholes (ID. 0.5 mm) distributed along the circumference to simulate the outflow pathway.

Before the experiment, the TOFC was filled with 8 mL of simulated HV. The central compartment of the cell was connected through the inlet port to the dispensing peristaltic pump (Minipuls^®^3, Gilson, Middleton, WI, USA) that provided a dynamic condition with a continuous aqueous flow of PBS (4.0 μL/min inflow). At starting time, an aliquot (200 μL) of BVZ 25 mg/mL was injected through the injection port at the bottom level in the simulated vitreous cavity of the cell. The injected dose of BVZ was about twice the standard clinical dose to account for the larger volume of this in vitro model. After the injection, at scheduled times up to 21 days, the aqueous fluid coming out from the TOFC via the outlet port of the anterior compartment was collected. Collected samples were stored at −20 °C prior to analysis for protein content by Bradford assay, a rapid and sensitive method used for measuring the concentrations of proteins. It is based on the shift in absorbance maximum of Coomassie brilliant blue G-250 dye from 465 to 595 nm following the binding to denatured proteins in the solution [24]. The experiment was repeated three times.

In a 96-well plate, 40 µL of each sample was mixed with 160 µL of Coomassie G-250 dye reagent solution diluted 1:4 with distilled water and incubated for 10 min at room temperature in the dark. Optical density was quantified at 595 nm in a plate reader. The readings were taken in triplicates and the mean and standard deviation were determined. The concentrations of unknown collected samples were estimated based on the standard curve derived from known concentrations of BVZ. Average measurements for blank (0 μg/mL of antibody) were subtracted from standards and unknown samples. Samples from each time point were evaluated in triplicate (*n* = 3) and the mean and standard deviation were determined.

#### 2.2.3. Cell Line Culture

The human pigmented retinal (ARPE-19) cell line (ATCC-CRL-2302) was cultured as monolayers in a complete medium consisting of DMEM-F12 medium supplemented with fetal calf serum (10%, *v*/*v*), L-glutamine (2 mM), penicillin (100 units/mL), and streptomycin (100 µg/mL). Cells were maintained in culture at 37 °C in a humidified atmosphere containing 5% CO_2_ in an incubator.

#### 2.2.4. Generation of 3D Cultures and Development of ARPE-19 Scaffold

For standard 2D cell cultures, 75 cm^2^ culture flasks were used. With the final aim of generating a 3D culture of ARPE-19, a commercial collagen porous scaffold of 12 mm diameter and 2 mm thickness was used. Each scaffold was first soaked overnight with a complete medium at 37 °C. Then, to understand the correct number of cells to seed over the scaffold, a first screening of different cell numbers for seeding was performed. Therefore, ARPE-19 cells in the exponential phase were detached using trypsin and counted, and different cell concentrations (2.5 × 10^5^, 5 × 10^5^, 1.0 × 10^6^) were seeded over collagen scaffold and maintained in culture at 37 °C. To avoid cell attachment to the plastic surface of plates, each scaffold was therefore distributed into a 24-weel plate, previously coated with a 1.5% of sterilized agarose (Sigma-Aldrich); then, ARPE-19 cells were seeded over the scaffold. ARPE-19 cells were left to grow over the scaffold for 6 days and the medium was changed every other day. At the end, scaffolds enriched with cells were washed with PBS and incubated with MTT solution for 3 h at 37 °C in order to color cells and to monitor their distribution over the scaffold.

#### 2.2.5. Setup of the TOFC Equipped with ARPE-19 Scaffold

ARPE-19 cells, grown in exponential phase in a plastic flask, were detached, counted, and 5 × 10^5^ cells were then seeded over the collagen scaffold, as previously described. After six days of cell seeding, the ARPE-19 scaffold was placed on grids and properly introduced between the central and posterior compartment. The three compartments were then assembled using screws and filled with simulated HV. Next, the inlet port of TOFC was connected to a dispensing peristaltic pump (Minipuls^®^ 3, Gilson, Middleton, WI, USA) via tubing (1.5 mm ID) to provide a continuous inflow (4.0 µL/min) of DMEM-F12, creating a dynamic condition referred to as d3D. To equilibrate the entire system, the ocular model equipped with ARPE-19 scaffold was left overnight in an incubator at 37 °C with 5% CO_2_. The day following the equilibration, 200 µL of BVZ (1 mg/mL in DMEM-F12) was injected with a needle at the bottom of the central compartment containing 8 mL of simulated HV, in order to obtain a 25 µg/mL BVZ final concentration in the TCOF. This chosen dose was necessary to maintain a drug concentration within the TOFC that was compatible with ARPE-19 cells. The TOFC was then left for 48 h in an incubator at 37 °C with 5% CO_2_ while being continuously perfused with DMEM-F12 at a 4.0 µL/min flow rate.

#### 2.2.6. Incubation of ARPE-19 Scaffold in Static Condition

To compare the effects of BVZ on ARPE-19 cells treated in 3D condition (d3D) derived from the ocular cell setup, a static condition (s3D) was also established as a reference. Therefore, three scaffolds per experiment were incubated with BVZ (25 µg/mL) for 48 h in an incubator at 37 °C with 5% CO_2_. Furthermore, the effect of BVZ treatment on scaffolds under s3D conditions was compared with that observed in untreated scaffolds under the same s3D conditions.

#### 2.2.7. Flow Cytometry Analysis and Evaluation of Cell Viability

BVZ activity was monitored cytofluorimetrically by evaluating VEGF fluorescent signal over the cells. Scaffold from s3D or d3D models, treated with BVZ for 48 h, or untreated scaffolds, i.e., untreated s3D and untreated d3D, underwent collagenase IV treatment at 37 °C for 20 min. The cells were collected using centrifugation and stained with the primary antibody VEGF (JH121) (1:500) in PBS for 1 h at room temperature. At the end of incubation, the samples were washed with PBS and then incubated with the secondary antibody goat anti-mouse IgG H&L (Alexa Fluor^®^ 647) and preabsorbed (1:2000) for 45 min at room temperature. At the end of the incubation, the cell pellet was washed with PBS and the sample was analyzed using a C6 flow cytometer (Accuri Cytometers, Milan, Italy). The analysis was performed by excluding cell debris based on forward scatter (FSC) and side scatter (SSC) parameters. VEGF fluorescent signal was then expressed as the integrated mean fluorescence intensity (iMFI), which represents the product of the frequency of VEGF positive cells and the mean fluorescence intensity of the cells. Moreover, to assess cell viability, digested cells from scaffolds, i.e., untreated s3D, s3D + BVZ, and untreated d3D and d3D + BVZ, were also incubated with propidium iodide (PI, 10 g/mL) for 20 min at 37 °C in PBS and then analyzed using a C6 flow cytometer. Three independent experiments were performed, with three replicates for each.

#### 2.2.8. Statistical Analysis

Data are shown as mean values ± standard deviation of three independent experiments. Statistical analyses were performed using Prism 9.0 software (GraphPad, La Jolla, CA, USA). According to the design of the experiment under analysis, multiple *t*-tests, and one-way ANOVA and Bonferroni’s tests were used to calculate the threshold of significance. The statistical significance threshold was set at *p* < 0.05.

## 3. Results

In the first part of the study, the in vitro ocular model (TOFC) was exploited to investigate the clearance profile of a monoclonal antibody, specifically BVZ, which is one of the most commonly used categories of IVT ophthalmic therapeutics.

**Figure 2:**
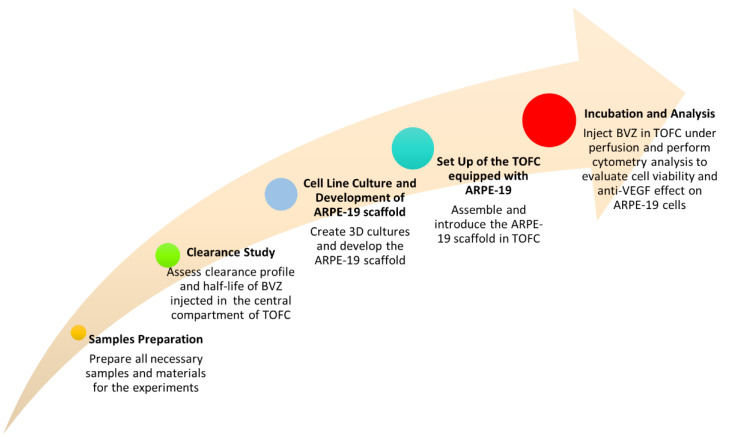
Flow chart representing the sequence of experiments performed to investigate the potential of TOFC equipped with ARPE-19 scaffold.

Afterwards, the work proceeded with the setup of ARPE-19 scaffold and its insertion into the TOFC in order to carry out cell viability studies and to investigate the anti-VEGF efficacy of BVZ under dynamic conditions of continuous perfusion. For the sake of clarity, the sequence of experiments conducted in this study is represented in Figure 2.

### 3.1. BVZ Clearance Study by TOFC

**Figure 3:**
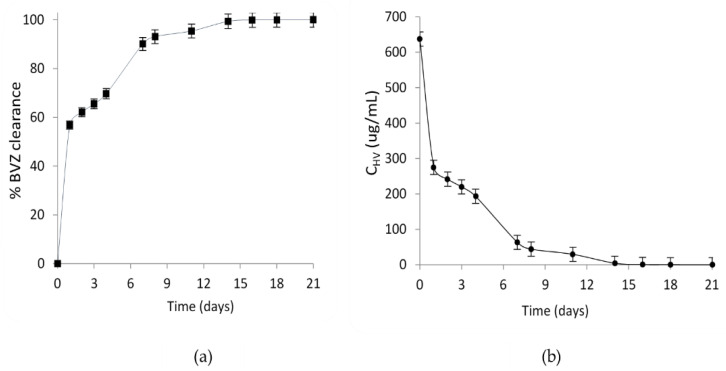
Graphical representation of BVZ (**a**) clearance profiles and (**b**) concentration in simulated HV after injection of BVZ (25 mg/mL) into the central compartment of the TOFC. Error bars in the graph represent the mean of three experiments.

To estimate the BVZ clearance profile, the outflow from the TOFC was collected by the outlet port of the anterior compartment over a period of 21 days. A graphical representation of clearance and concentration profiles is provided in Figure 3a,b, respectively.

The clearance profile, as depicted in Figure 3a, exhibited a first-order pattern characterized by a rapid initial increase followed by a more gradual upsurge. This behavior suggests a biphasic elimination process, possibly involving both saturable and non-saturable clearance mechanisms. These findings align with the known pharmacokinetic complexity of large molecular weight compounds.

In Figure 3b, the concentration profile shows a corresponding trend, indicating a decline in BVZ concentration in the HV over the observation period. Notably, the initial steep decline in concentration mirrored the rapid clearance phase observed in the clearance profile. The half-life of BVZ in TOFC was calculated to be 1.5 ± 0.4 days, underscoring the rapid elimination phase of the drug.

### 3.2. Development of ARPE-19 Scaffold and Equipping within TOFC

**Figure 4:**
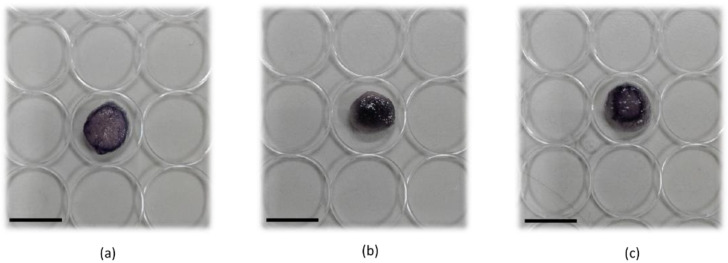
ARPE-19 cell distribution on collagen scaffold. ARPE-19 cells were grown over the collagen scaffold for 6 days and then scaffolds were incubated with MTT to stain cell distribution. Representative images of different cell seeding amounts: (**a**) 2.5 × 10^5^; (**b**) 5.0 × 10^5^; (**c**) 1.0 × 10^6^. Scale bar: 10 mm.

The correct distribution of cells inside the scaffold support represents a crucial aspect to determine the initial cellular setup required for each in vitro experiment. For this reason, at the end of 6 days of culture over the scaffold, the distribution of ARPE-19 cells in the scaffold was monitored using coloring cells with MTT solution (Figure 4). A homogeneous distribution and growth of the cells was observed in the scaffold with 5.0 × 10^5^ cells seeded (Figure 4b), while, when 2.5 × 10^5^ cells were seeded, just a peripheral distribution of the cells was observed (Figure 4a), like a 1.0 × 10^6^ cell seeding condition (Figure 4c). Consequently, for further experiments, 5 × 10^5^ was chosen as the starting seeding number of cells on the collagen scaffold.

**Figure 5:**
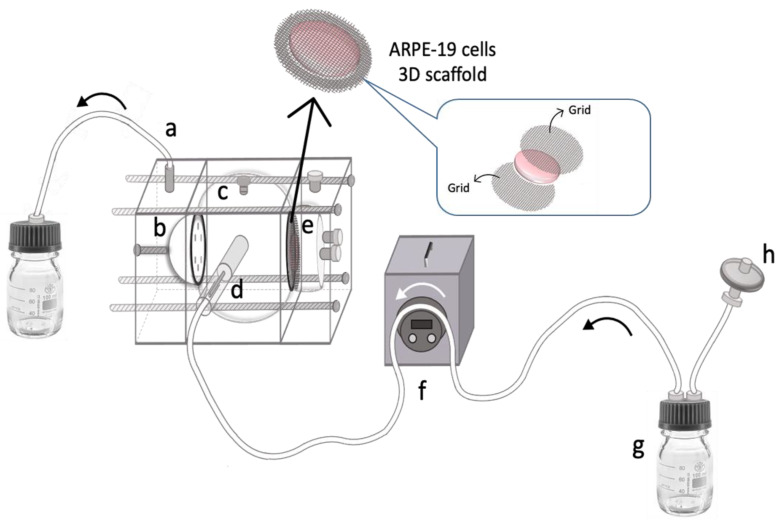
Schematic representation of the TOFC equipped with ARPE-19 scaffold. The model comprises: (a) an outlet port allowing outflow from the anterior compartment; (b) an 8-microhole perforated disk between the anterior and central compartment; (c) an injection port at the top of the central compartment; (d) an inlet port connected to a (f) peristaltic pump; (e) ARPE-19 scaffold vertically positioned between the central and the posterior compartment; (g) a reservoir of DMEM-F12 and (h) a sterile filter with 0.22 µm pore size.

Six days after the seeding, the ARPE-19 scaffold was vertically positioned between the central and the posterior compartment of the TOFC in order to simulate the retinal blood barrier. The three compartments were then assembled and connected to the peristaltic pump, as represented in Figure 5.

As shown in Figure 5, the developed ARPE-19 scaffold is placed between the central and posterior compartments and is supported by two grids to keep it in a vertical position. The inlet port is connected to a peristaltic pump via tubing (1.5 mm ID), providing continuous infusion of the completed cell culture medium (4.0 µL/min) to the area near the ARPE-19 scaffold. The aqueous outflow is collected from the anterior cavity via the outlet port, which is elevated to approximately 3.0 cm to maintain a full internal volume within the model by providing a small amount of back pressure. The overall size and volume of TOFC are approximately twice that of the human eye to facilitate analysis of drug distribution and pharmacokinetics in the ocular environment. Indeed, creating a model that is larger than the human eye allows for better visualization and analysis of drug behavior in the vitreous.

### 3.3. Evaluation of Anti-VEGF Activity and Cell Viability

To assess the effectiveness of BVZ in inhibiting VEGF, ARPE-19 scaffold cultured under dynamic (d3D) conditions was analyzed 48 h after BVZ injection (0.2 mL, 1 mg/mL) in the central compartment of the TOFC. The results were further compared to both untreated d3D conditions and also versus ARPE-19 cell growth in an s3D condition or s3D incubated with BVZ at the same concentration (25 µg/mL).

**Figure 6:**
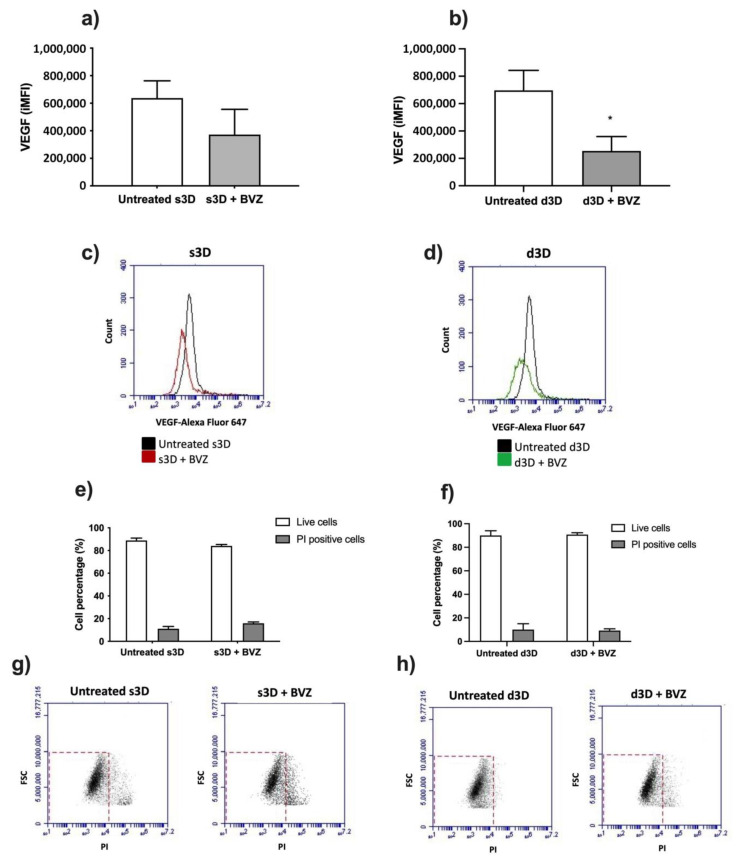
Evaluation of BVZ activity on VEGF. ARPE-19 cells grown under static (s3D; (**a**,**c**,**e**,**g**)) or dynamic condition (d3D; (**b**,**d**,**f**,**h**)) were left untreated or underwent BVZ treatment (25 µg/mL) for 48 h; then cytofluorimetric evaluation of VEGF on cells derived from scaffold was performed. VEGF fluorescent signal in ARPE-19 cells is shown as the integrated mean fluorescence (iMFI; (**a**,**b**)) along with representative flow cytometry plots (**c**,**d**). Statistically significant versus untreated condition d3D (Ctrl): * *p* < 0.05, *n* = 3). Propidium iodide (PI) staining is shown as positive cell percentage in s3D (**e**) or d3D (**f**) along with representative flow cytometry plots (**g**,**h**). Data are expressed as the percentage of cells ± standard deviation (no statistical significance of BVZ-treated scaffold versus untreated condition, *n* = 3).

This approach allowed for a comprehensive evaluation of BVZ activity and its impact on the ARPE-19 scaffold under different conditions. According to data obtained by flow cytometry, BVZ was able to reduce the VEGF fluorescent signal both in s3D and d3D (Figure 6a,b); it is the reduction that is only statistically significant (*p* < 0.05) in d3D conditions (Figure 6b) when compared to the respective untreated condition. Moreover, from the comparison of the VEGF fluorescence signal between s3D and d3D, it was discovered to be not statistically significant.

Moreover, the PI evaluation of the two considered scaffold conditions showed no statistically significant difference compared to the untreated scaffold conditions, showing that the BVZ concentration used did not affect cell viability over the scaffold (Figure 6e,f), as is also appreciated by the representative cytofluorimetric dot plots (Figure 6g,h).

## 4. Discussion

The development of a tricompartmental ocular flow model, namely the TOFC, arose from the need to find alternative models that enable the occurrence of pharmacokinetic and pharmacodynamic studies while limiting the use of animal experimentation during the initial development phase of new IVT ophthalmic therapies. After all, the search for alternative methods to animal experimentation is a topic of increasing interest that applies to the development of new therapeutic systems as a whole and arises from the need to respond to important ethical concerns. In vitro models have proven valuable for their ability to mimic physiological patterns, permitting the study of the safety and efficacy of potential therapeutics before they are tested in animal models or in human subjects [2].

In our previous research, TOFC was employed to assess the release profile and clearance of cefuroxime delivered within a nanoparticle system [22], whereas in this study, the same model was utilized to investigate the clearance of BVZ IVT administered as an anti-VEGF agent for a variety of ocular disorders.

The commercial solution of BVZ was injected without dilution at twice the standard clinical dose (5.0 mg BVZ; 0.2 mL) to account for the model’s large volume. This dosage adjustment was necessary to ensure that the drug concentration inside the TCOF was comparable to that of the human eye and to enable the accurate analysis of drug distribution in the ocular environment and outflow.

The biphasic trend in the BVZ clearance profile observed in this study finds support in the literature. Ahn et al. compared the clearance of IVT-injected BVZ in vitrectomized versus non-vitrectomized control rabbit eyes. In both of the experimental groups, they observed a two-phase elimination pattern with a first fast distribution phase, followed by a second slow elimination phase [25].

The short half-life (1.5 ± 0.4 days) extrapolated by the clearance profile corresponds well with the steep initial decline in concentration observed in the concentration profile, providing a quantitative measure to the observed trend. This relatively short half-life suggests that a significant proportion of BVZ is cleared from the system within a short period after administration, necessitating the careful consideration of dosing frequency to maintain therapeutic efficacy.

Several in vivo studies analyzed the pharmacokinetic parameters of IVT-administered BVZ in different animal eye models, mainly rats, rabbits, or monkeys. It was encouraging that the BVZ half-life value found in this study is quite similar to that found by Gal-Or and colleagues [26], who investigated and characterized the presence of IVT-injected BVZ in the aqueous outflow channels of a rat model. According to their study, BVZ molecules passed through the aqueous outflow channels within 48 h following IVTl injection. In contrast, Nomoto et al. and Sinapis et al. [27,28] found the half-life of BVZ in the vitreous humor after IVT injection to be 6–6.61 days in rabbit eyes. In human eyes, at a similar dose, the half-life of BVZ is reported to be 4.9 days or 0.66 days in non-vitrectomized or vitrectomized eyes, respectively [29].

It is evident that the half-life of IVT-injected BVZ can vary significantly across different studies, mainly due to the specific experimental models employed and varying conditions such as vitrectomized versus non-vitrectomized eyes, and lentomized versus non-lentomized eyes. Therefore, given this variability, making direct comparisons among these studies may not be entirely appropriate.

Furthermore, it is important to emphasize that while in vitro experiments provide valuable insights, they cannot completely replace preclinical in vivo models. Instead, they should be considered as complementary tools, particularly useful for initial screening when selecting among various formulations those that warrant further investigation.

Beyond assessing the suitability of TOFC for studying IVT drug clearance, the primary emphasis of this study lies in introducing a novel element: a retinal cellular scaffold. Specifically, a scaffold of ARPE-19 cells that has been developed and positioned between the central and posterior compartments to partially simulate the retina. This allows for the creation of an artificial retina model that can be maintained in culture through the dynamic condition created by the peristaltic pump. Overall, the ocular flow cell equipped with the inserted ARPE-19 scaffold enables not only the assessment of resident time and drug clearance profile, but also the evaluation of the activity of drugs, whether they are free or entrapped in a drug delivery system.

Various in vitro models have been proposed in the literature to evaluate and describe drug distribution in the vitreous, but to the best of our knowledge, none of them have previously included a retinal cell culture scaffold in an assembled compartmentalized ocular model. Furthermore, most of the 3D models proposed take into consideration the realization of 3D models of the cornea, even if they are still far from the complex equipment of the eye, like for example, the lacrimal apparatus responsible for cleansing and supporting regeneration [30,31].

Recently, few works reported the use of alternative in vitro 3D models for ocular investigation. Auel and colleagues developed a technique to continuously observe the distribution of the drug in in vitro vitreous substitutes by using a 3D-printed device that allows the injection volume of 100 µL, which corresponds to a commonly therapeutically injected volume of IVT injection in vivo. This system therefore allows a reduction in the number of necessary animals in preclinical studies of new IVT dosage forms [32]. The use of 3D models in in vitro experimentations allows the recreation and maintenance of a suitable microenvironment to support cell growth and function, reducing the wide gap between in vitro and in vivo experimentations [7]. Among tissue engineering, porous 3D scaffolds are commonly used in several applications and can be made from various materials, according to the need and the type of pores required. In particular, porous scaffold networks that enable the transport of nutrients, removal of wastes, and facilitate the proliferation and migration of cells are essential. The porosity and pore size influences cell behavior and determines the final mechanical property of the scaffold [33].

Therefore, keeping these objectives in mind, the ARPE-19 scaffold was successfully developed and installed in the in vitro ocular model proposed in this study (Figure 5).

Several in vitro studies on ocular cells in culture, such as human retinal pigment epithelium, optic nerve head astrocytes, and human corneal cells, have been reported on the effect of BVZ [34,35]. BVZ has the main role to bind all circulating VEGF-A isoforms. By binding to VEGF-A, it prevents the interaction of VEGF-A with VEGFR and thereby inhibits the activation of VEGF signaling pathways that promote neovascularization [36]. Even though IVT injection uses minute amounts of the drug, the full-sized antibody, BVZ, has more potential to cause inflammatory and immune reactions over time. To avoid possibly severe systemic side effects, the treatment of ocular disease is usually limited to intraocular administration, in which case retinal toxicity is the primary concern [35]. Specifically, in the work of Kaempf and colleagues, neural retinal specimens were placed on nitrocellulose membranes and transferred to a sterile cell carrier under a perfusion system at 1 mL/h. The system was then treated with different concentrations of BVZ (0.25 mg/mL, 0.5 mg/mL, and 1.25 mg/mL). Authors underlined that in their model, BVZ was well tolerated by cells even if possible side effects on mature vessels had to be considered and may explain the higher risk for cardiovascular events in anti-VEGF treatments.

In support of our data, Chung and colleagues [37] developed an organotypic eye-on-a-chip model that mimics the retinal pigment epithelium (RPE)–choroid complex in vitro. This model consists of an RPE monolayer and adjacent perfusable blood vessel network, which supports the barrier function of the outer blood–retinal barrier. Treatment with BVZ caused a regression in the vessel network, demonstrating a significant antiangiogenic effect, confirming the main characteristic of the model to be used to evaluate not only the cytotoxic activity of a drug, but also to determine the appropriate drug dosage.

In the present study, a simplified prototype scaffold composed of a unique cell line (ARPE-19) was developed and employed. The selection of this specific cell line was based on the anatomical positioning of retinal pigment epithelium beneath neural cells within the retina, as well as its integral role as the initial component of the tri-layered structure constituting the blood–retinal barrier, which includes the retinal pigmented epithelium, Bruch’s membrane, and the vascular endothelium. Furthermore, the suitability of the ARPE-19 cell line for our investigations is underscored by its inherent ability to secrete vascular endothelial growth factor (VEGF) under normal physiological conditions [38]. This characteristic enabled us to assess the potential of this in vitro model for evaluating the anti-VEGF activity of BVZ.

Certainly, our study is not devoid of limitations. Firstly, like many other in vitro models designed to replicate the cellular and tissue environments of living organisms, our TOFC model should be interpreted with caution, as it inherently offers only a partial representation of real biological systems. Despite our efforts to faithfully recreate the ocular system and its existing barriers, our model does lack certain anatomical components, such as the lens. It is worth noting that the literature reports underscore the significant contribution of the lens to ocular pharmacokinetics, with one study indicating a substantial reduction in BVZ half-life in lensectomized eyes [39].

Another limitation that we aim to overcome in the future is related to the cellular scaffold. We are hopeful that in the near future we can develop a more realistic and representative model of the blood–retinal membrane by co-culturing retinal epithelial cells with retinal endothelial cells within the same 3D scaffold, as well as incorporating injury insults that induce retinal disease and loss of visual function. This approach should allow us to better mimic the complex interactions and barriers present in the ocular system, further improving the accuracy and applicability of our model for ocular pharmacokinetic studies.

In forthcoming research, in order to enhance our model, it will be imperative to consider the employment of alternative materials to Plexiglass and explore the potential utilization of 3D printing technology. 3D printing offers a more versatile and continually advancing technology within the biomedical field, which could provide us with a platform to construct more anatomically comprehensive and functionally representative ocular models.

## 5. Conclusions

In the present study, we demonstrated and confirmed that the TOFC is a new and promising in vitro ocular model with great potential. By developing a 3D ARPE-19 scaffold that partially mimics the presence of the retina, we were able to provide a more accurate representation of drug distribution in the vitreous cavity, as well as its biological activity. This information is crucial in the development of novel therapeutic strategies for posterior eye conditions.

As previously demonstrated [22], the TOFC proposed here has the capability of simulating the human eye environment, enabling the investigation of the clearance profile without the use of animal experimentation in the early stages of developing new ophthalmic therapeutics. This is important as it reduces the reliance on animal experimentation and can lead to faster and more ethical drug development.

Overall, the findings suggest that the ocular flow model equipped with ARPE-19 scaffold described in this study can be considered a promising platform for investigating pharmacokinetics and the activity of new ophthalmic formulations intended for the treatment of the posterior eye segment. Further studies are warranted to confirm these results and to explore the full potential of this in vitro model.

## Data Availability

Not applicable.

## References

[1] Christensen G., Barut L., Urimi D., Schipper N., Paquet-durand F. (2021). Investigating Ex Vivo Animal Models to Test the Performance of Intravitreal Liposomal Drug Delivery Systems. Pharmaceutics.

[2] Lehrmann D., Refaian N., Simon M., Rokohl A.C., Heind L.M. (2022). Preclinical models in ophthalmic oncology—A narrative review. Ann. Eye Sci..

[3] 2021/2784(RSP). https://oeil.secure.europarl.europa.eu/oeil/popups/ficheprocedure.do?lang=en&reference=2021/2784.

[4] Gan J., Bolon B., Van Vleet T., Wood C., Haschek W.M., Rousseaux C.G., Wallig M.A., Bolon B. (2022). Chapter 24—Alternative Models in Biomedical Research: In Silico, In Vitro, Ex Vivo, and Nontraditional In Vivo Approaches. Haschek and Rousseaux’s Handbook of Toxicologic Pathology.

[5] Verderio P., Lecchi M., Ciniselli C.M., Shishmani B., Apolone G., Manenti G. (2023). 3Rs Principle and Legislative Decrees to Achieve High Standard of Animal Research. Animals.

[6] Mengus C., Muraro M.G., Mele V., Amicarella F., Manfredonia C., Foglietta F., Muenst S., Soysal S.D., Iezzi G., Spagnoli G.C. (2018). In Vitro Modeling of Tumor−Immune System Interaction. ACS Biomater. Sci. Eng..

[7] Foglietta F., Canaparo R., Muccioli G., Terreno E., Serpe L. (2020). Methodological aspects and pharmacological applications of three-dimensional cancer cell cultures and organoids. Life Sci..

[8] Hirt C., Papadimitropoulos A., Muraro M.G., Mele V., Panopoulos E., Cremonesi E., Ivanek R., Schultz-Thater E., Droeser R.A., Mengus C. (2015). Bioreactor-engineered cancer tissue-like structures mimic phenotypes, gene expression profiles and drug resistance patterns observed “in vivo”. Biomaterials.

[9] Fotaki N. (2011). Flow-through cell apparatus (USP apparatus 4): Operation and features. Dissolut. Technol..

[10] Tojo K. (2004). A pharmacokinetic model for ocular drug delivery. Chem. Pharm. Bull..

[11] Repetto R., Stocchino A., Cafferata C. (2005). Experimental investigation of vitreous humour motion within a human eye model. Phys. Med. Biol..

[12] Awwad S., Lockwood A., Brocchini S., Khaw P.T. (2015). The PK-Eye: A Novel in Vitro Ocular Flow Model for Use in Preclinical Drug Development. J. Pharm. Sci..

[13] Adrianto M.F., Annuryanti F., Wilson C.G., Sheshala R., Thakur R.R. (2022). In vitro dissolution testing models of ocular implants for posterior segment drug delivery. Drug Deliv. Transl. Res..

[14] Loch C., Nagel S., Guthoff R., Seidlitz A., Weitschies W. (2012). The Vitreous Model—A new in vitro test method simulating the vitreous body Model characterization. Biomed. Eng. /Biomed. Tech..

[15] Loch C., Bogdahn M., Stein S., Nagel S., Guthoff R., Weitschies W., Seidlitz A. (2014). Simulation of Drug Distribution in the Vitreous Body After Local Drug Application into Intact Vitreous Body and in Progress of Posterior Vitreous Detachment. J. Pharm. Sci..

[16] Stein S., Auel T., Kempin W., Bogdahn M., Weitschies W., Seidlitz A. (2018). Influence of the test method on in vitro drug release from intravitreal model implants containing dexamethasone or fluorescein sodium in poly (D,L-lactide-co-glycolide) or polycaprolactone. Eur. J. Pharm. Biopharm..

[17] Auel T., Großmann L., Schulig L., Weitschies W., Seidlitz A. (2021). The EyeFlowCell: Development of a 3D-Printed Dissolution Test Setup for Intravitreal Dosage Forms. Pharmaceutics.

[18] Yang X., Guo X., Yang Y., Huang J., Xiong X., Xie X., Tan X. (2009). In-Vitro Eyeball Superfusion System. Patent.

[19] Awwad S., Bouremel Y., Ibeanu N., Brocchini S.J., Khaw P.T. (2021). Artificial Eye Assembly for Studying Ocular Pharmacokinetics. Patent.

[20] Juhong H., Yambin P. (2020). Medical Simulation Human Eye Simulation Module. Patent.

[21] Dongeun H., Jeongyun S. (2017). Methods and Devices for Modelling the Eye. Patent.

[22] Sapino S., Peira E., Chirio D., Chindamo G., Guglielmo S., Oliaro-Bosso S., Barbero R., Vercelli C., Re G., Brunella V. (2019). Thermosensitive nanocomposite hydrogels for intravitreal delivery of cefuroxime. Nanomaterials.

[23] Kummer M.P., Abbott J.J., Dinser S., Nelson B.J. Artificial vitreous humor for in vitro experiments. Proceedings of the Annual International Conference of the IEEE Engineering in Medicine and Biology.

[24] Bradford M.M. (1976). A rapid and sensitive method for the quantitation of microgram quantities of protein utilizing the principle of protein-dye binding. Anal. Biochem..

[25] Ahn J., Kim H., Woo S.J., Park J.H., Park S., Hwang D.J., Park K.H. (2013). Pharmacokinetics of intravitreally injected bevacizumab in vitrectomized eyes. J. Ocul. Pharmacol. Ther..

[26] Gal-Or O., Dotan A., Dachbash M., Tal K., Nisgav Y., Weinberger D., Ehrlich R., Livnat T. (2016). Bevacizumab clearance through the iridocorneal angle following intravitreal injection in a rat model. Exp. Eye Res..

[27] Nomoto H., Shiraga F., Kuno N., Kimura E., Fujii S., Shinomiya K., Nugent A.K., Hirooka K., Baba T. (2009). Pharmacokinetics of Bevacizumab after Topical, Subconjunctival, and Intravitreal Administration in Rabbits. Investig. Ophthalmol. Vis. Sci..

[28] Sinapis C.I., Routsias J.G., Sinapis A.I., Sinapis D.I., Agrogiannis G.D., Pantopoulou A., Theocharis S.E., Baltatzis S., Patsouris E., Perrea D. (2011). Pharmacokinetics of intravitreal bevacizumab (Avastin^®^) in rabbits. Clin. Ophthalmol..

[29] Moisseiev E., Waisbourd M., Ben-Artsi E., Levinger E., Barak A., Daniels T., Csaky K., Loewenstein A., Barequet I.S. (2014). Pharmacokinetics of bevacizumab after topical and intravitreal administration in human eyes. Graefes Arch. Clin. Exp. Ophthalmol..

[30] Tegtmeyer S., Papantoniou I., Müller-Goymann C.C. (2001). Reconstruction of an in vitro cornea and its use for drug permeation studies from different formulations containing pilocarpine hydrochloride. Eur. J. Pharm. Biopharm..

[31] Kutlehria S., Sachdeva M.S. (2021). Role of In Vitro Models for Development of Ophthalmic Delivery Systems. Crit. Rev. Ther. Drug Carrier Syst..

[32] Auel T., Scherke L.P., Hadlich S., Mouchantat S., Grimm M., Weitschies W., Seidlitz A. (2023). Ex Vivo Visualization of Distribution of Intravitreal Injections in the Porcine Vitreous and Hydrogels Simulating the Vitreous. Pharmaceutics.

[33] Loh Q.L., Choong C. (2013). Three-dimensional scaffolds for tissue engineering applications: Role of porosity and pore size. Tissue Eng. Part B Rev..

[34] Merz P.R., Röckel N., Ballikaya S., Auffarth G.U., Schmack I. (2018). Effects of ranibizumab (Lucentis^®^) and bevacizumab (Avastin^®^) on human corneal endothelial cells. BMC Ophthalmol..

[35] Kaempf S., Johnen S., Salz A.K., Weinberger A., Walter P., Thumann G. (2008). Effects of Bevacizumab (Avastin) on Retinal Cells in Organotypic Culture. Investig. Ophthalmol. Vis. Sci..

[36] Ferrara N., Hillan K.J., Gerber H.P., Novotny W. (2004). Discovery and development of bevacizumab, an anti-VEGF antibody for treating cancer. Nat. Rev. Drug Discov..

[37] Chung M., Lee S., Lee B.J., Son K., Jeon N.L., Kim J.H. (2018). Wet-AMD on a Chip: Modeling Outer Blood-Retinal Barrier In Vitro. Adv. Healthc. Mater..

[38] Ma W., Lee S.E., Guo J., Qu W., Hudson B.I., Schmidt A.M., Barile G.R. (2007). RAGE ligand upregulation of VEGF secretion in ARPE-19 cells. Investig. Ophthalmol. Vis. Sci..

[39] Christoforidis J.B., Williams M.M., Wang J., Jiang A., Pratt C., Abdel-Rasoul M., Hinkle G.H., Knopp M.V. (2013). Anatomic and pharmacokinetic properties of intravitreal bevacizumab and ranibizumab after vitrectomy and lensectomy. Retina.

